# The impact of antibiotic use on clinical features and survival outcomes of cancer patients treated with immune checkpoint inhibitors

**DOI:** 10.3389/fimmu.2022.968729

**Published:** 2022-07-28

**Authors:** Jiaxin Zhou, Guowei Huang, Wan-Ching Wong, Da-hai Hu, Jie-wen Zhu, Ruiman Li, Hong Zhou

**Affiliations:** ^1^ Department of Obstetrics and Gynecology, The First Affiliated Hospital of Jinan University, Guangzhou, China; ^2^ International School, Jinan University, Guangzhou, China; ^3^ Shunde Hospital Affiliated to Jinan University, Guangzhou, China; ^4^ Department of General Surgery, The First Affiliated Hospital of Jinan University, Guangzhou, China; ^5^ College of Science and Engineering, Jinan University, Guangzhou, China

**Keywords:** antibiotic, immune checkpoint inhibitor, PD-1, PD-L1, survival outcomes

## Abstract

**Background:**

Nowadays, immune checkpoint inhibitors (ICIs) have become one of the essential immunotherapies for cancer patients. However, the impact of antibiotic (ATB) use on cancer patients treated with ICIs remains controversial.

**Methods:**

Our research included retrospective studies and a randomized clinical trial (RCT) with cancer patients treated with ICIs and ATB, from the public database of PubMed, Web of Science, Embase, Cochrane, clinical trials, and JAMA. The survival outcomes included progression-free survival (PFS) and overall survival (OS). Meanwhile, hazard ratios (HRs) and 95% confidence intervals (CIs) were calculated, and subgroup analyses were performed to determine the concrete association between ATB use and the prognosis of cancer patients treated in ICIs.

**Results:**

Our results revealed that ATB use was associated with poor survival outcomes, including OS (HR: 1.94, 95% CI: 1.68–2.25, p <0.001) and PFS (HR: 1.83, 95% CI: 1.53–2.19, p <0.001). The subgroup analysis learned about the association between ATB use and the prognosis of cancer patients with ICI treatment, including 5 cancer types, 3 kinds of ICI, 5 different ATP windows, broad-spectrum ATB class, and ECOG score. ATB treatment was associated with poor OS of non-small-cell lung cancer (NSCLC), renal cell carcinoma (RCC), esophageal cancer (EC), and melanoma (MEL) in patients treated in ICIs, while non-small-cell lung cancer (NSCLC) and renal cell carcinoma (RCC) were associated with poor PFS. Meanwhile, it was strongly related to the ICI type and ATB window. Furthermore, it is firstly mentioned that the use of broad-spectrum ATB class was strongly associated with poor PFS.

**Conclusion:**

In conclusion, our meta-analysis indicated that ATB use was significantly associated with poor OS and PFS of cancer patients treated with ICI immunotherapy, especially for patients with ATB use in the period of (−60 days; +30 days) near the initiation of ICI treatment. Also, different cancer types and the ICI type can also impact the survival outcome. This first reveals the strong relationship between the broad-spectrum ATB class and poor PFS. Still, more studies are needed for further study.

## Introduction

Working *via* the anti-tumor immune response, immune checkpoint inhibitors (ICIs) have proved a promising therapeutic treatment in the clinic, which was designed to interfere with inhibitory pathways that naturally constrain T cell reactivity (1). ICIs reinvigorate anti-tumor immune responses by disrupting co-inhibitory T-cell signaling (2). In the last decade, ICIs have caused a major paradigm shift in cancer therapy. It has been approved for various cancers and has improved the survival outcome for many patients (3). However, although ICIs did improve the survival outcome of cancer treatment, the efficacy of the ICI drugs is still limited due to refractiveness, and there are still some uncertain points regarding ICI therapy (4). Additionally, the use of ICIs can induce unique side effects called immune-related adverse events, which can vary a lot in different individuals (5). Some patients exhibit an atypical treatment response pattern with new or enlarging lesions, which needs further observation to determine the process (6). The side effects of ICI therapy involve various organs and systems, including the thyroid and pituitary glands, skin, and digestive system and respiratory system, which can markedly affect the physiological function of organs and the quality of life of patients, even causing fatal consequences in some extreme cases (7). Thus, it is urgent and necessary to find the novel biomarkers to select the patients who can most benefit from the drugs that are in need of being identified.

Antibiotic (ATB) therapy has produced indispensable advances for patients with cancer, populations who are more easily get infected by bacterial because of treatment-related immune suppression. The derangement of the gut microbiota environment has been increasingly well-characterized because of the existence of tumor-specific immune tolerogenesis (8). However, the association between ATB use and the prognosis of cancer patients in ICI treatment remains controversial. Some studies have reported that antibiotic use can result in reduced efficacy of immune checkpoint inhibitors, which can be the consequence of dysbiosis of the intestinal microbiome, a main determinant of the cancer-immune set point of patients (9). Meanwhile, the perturbation of the gut microbiota has been indicated as a possible mechanism to explain the adverse effects attributed to antibiotic exposure in the context of ICI therapy (10). Some studies have found that exposure to antibiotic therapy can influence the probability of response to ICI and predict worse patient survival across malignancies (11). However, ATB use can eliminate the infection and improve the quality of infected patients. Therefore, it is necessary to determine whether ATB use affects the efficacy of ICI treatment and the prognosis of cancer patients.

To learn about the specific association between ATB use and ICI treatment of cancer patients and provide potential reference to clinic performance, the current meta-analysis was performed to clarify if ATB use will impact the survival outcome of cancer patients treated in ICIs, and whether any clinical factors could be used to predict the response of patients to ICIs.

## Materials and methods

### Literature searching strategy

Our meta-analysis protocol was submitted to the International Prospective Register of Systematic Reviews (PROSPERO CRD 42022330156), and this research followed the Preferred Reporting Items for Systematic Reviews and Meta-Analyses guidelines. Electronic databases including PubMed, Web of Science, Embase, Cochrane Library, and Clinical Trials were searched using MeSH words obtained from the National Center for Biotechnology Information (NCBI). Furthermore, the reference lists of eligible reports were also searched to identify potentially relevant studies (“Antibiotics, Antitubercular” AND “Antibiotics, Antineoplastic” AND “Anti-Bacterial Agents”) AND (“Immune Checkpoint Inhibitors” OR “Immune Checkpoint Inhibitors” (Pharmacological Action) OR “Immune Checkpoint Proteins”) were used as the search query.

### Inclusion and exclusion criteria

These criteria were developed by all the authors. Inclusion criteria: (I) publications studied the ATB use in cancer patients with ICI treatment; (II) patients were divided into different groups, according to whether they were treated with ATB; (III) the studies should include standard and sufficient data; (IV) research data must be obtained independently by relative organizations; and (V) the publication language was English. Exclusion criteria: (I) duplicate publications and data; (II) relevant research data in the literature comes from public databases; (III) literature types are reviews, case reports, meeting abstracts, and basic experimental research literature; and (IV) literature language is not English.

### Data extracting and quality assessment

From each of the included literature, the following data were collected: the name of author, publication year, country or area, the number of patients, study design, cancer type, ICI treatment, antibiotic treatment information (ATB window and drug type), median OS, median PFS, survival outcome (OS and PFS). Meanwhile, to learn about the concrete relationships between ATB treatment and clinical features of ICI-treated cancer patients, the baseline characteristics of patients, including gender, ICI line, cancer stage grade, and lung cancer, were also recorded. These data were reported in a standardized data extraction spreadsheet for further analysis. The quality assessment of eligible studies was done independently using the Newcastle–Ottawa scale.

### Statistical analysis

The meta-analysis was conducted to calculate the pooled HRs with corresponding 95% Confidential Intervals (CIs) by using Review Manager 5.4 software for Mac. To avoid the potential heterogeneity affection, a random-effects model was chosen to analyze the survival outcome. Moreover, the dichotomous and generic inverse variance method models were adopted to analyze the extracted data. Statistical heterogeneity was assessed using the χ^2^ test and the I^2^ test, and publication bias was assessed by funnel plots. Statistical significance was considered in this study when *p <*0.05.

## Results

### Study selection

**Figure 1:**
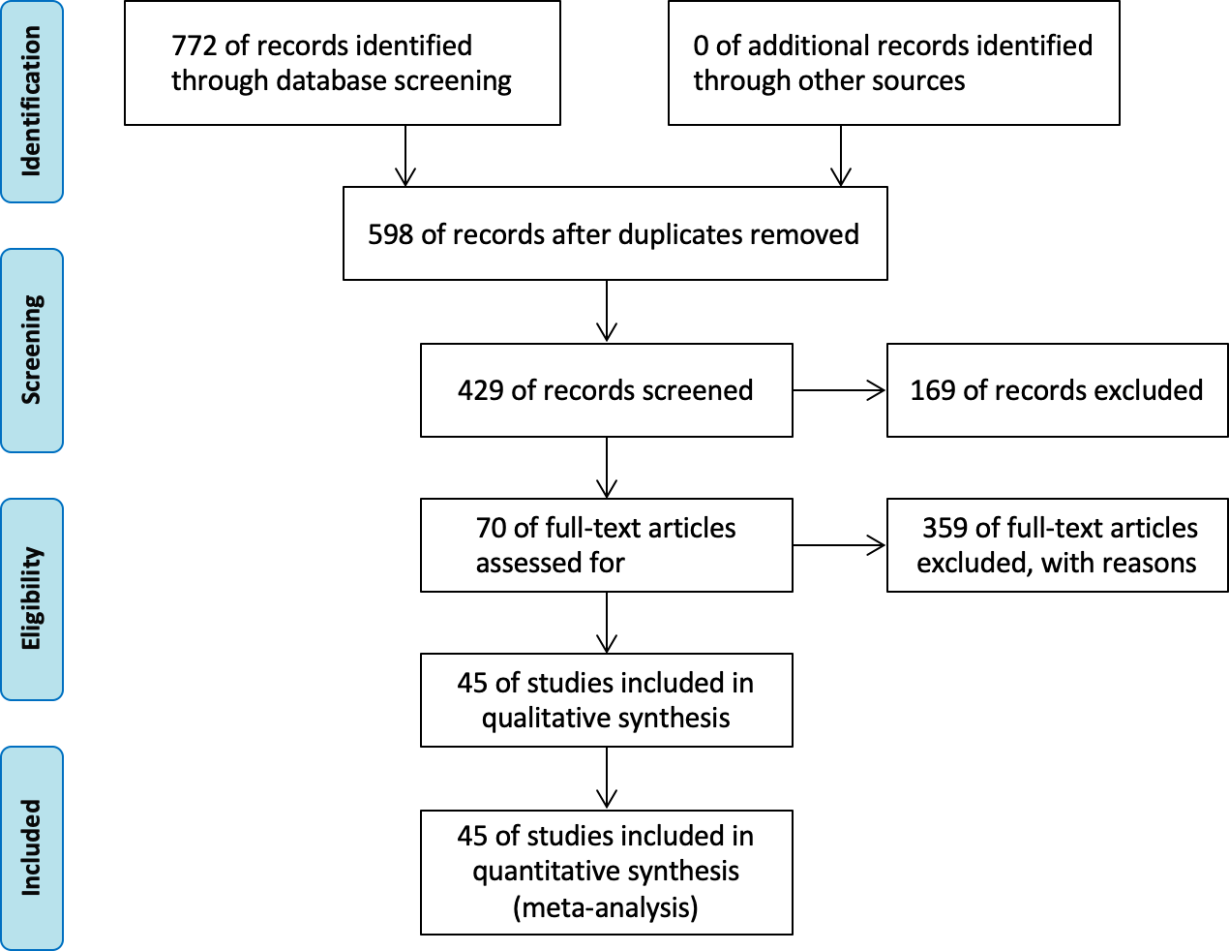
Flow diagram of the study search and selection in this meta-analysis.

The initial literature search identified 772 reports, including 409 reports from PubMed, 188 reports from the Web of Science, 103 reports from the Embase database, 24 from the Cochrane database, 30 from clinical trials, and 18 from the JAMA database. After removing duplicate reports, 678 pieces of literature were considered potentially eligible. Finally, according to the above including and excluding criteria, 45 articles were selected, including 12,493 patients. The survival outcomes were composed of progress-free survival outcomes (PFS) and overall survival (OS). The study selection flowchart isshown in **Figure 1**.

### Baseline characteristics of included studies

The eligible studies included 12,493 patients and 13 kinds of cancer types: lung cancer, head and neck cancers, renal cell carcinoma, acute myelocytic leukemia, melanoma, urothelial carcinoma, esophageal squamous cell carcinoma, liver cancer, porocarcinoma, digestive tract carcinoma, Hodgkin’s lymphoma, cervical cancer, and cholangiocarcinoma. The publication year ranged from 2017 to 2022, and the studies were performed in 17 different areas. Among all the studies, five of them used randomized controlled trial (RCT) designs, while other studies were retrospective. Seventeen of the studies used only one kind of ICI, including PD-1 (programmed cell death protein 1) inhibitor, PD-L1 (programmed cell death 1 ligand 1) inhibitor, and CTLA-4 (cytotoxic T-lymphocyte-associated protein 4) inhibitor (PD-1 inhibitors: n = 12; PD-L1 inhibitors: n = 4; CTLA-4 inhibitors: n = 1). While four of them were not definite, the ICI therapy and the other research used a combination of different ICI treatments for at least two of them. The detailed information is shown in **Table 1**.

**Table 1:** Basic characteristics of the studies included in the meta-analysis (n = 45).

Study	Year	Patients	Area	ICI type	ATB window	Method
A. Iglesias‐Santamariía (12)	2019	102	Spain	CTLA-4, PD-1,PD-L1	(−28,28)	Retrospective cohort study	
Akhil Kapoor (13)	2020	155	India	nivolumab	(−14,14)	Retrospective cohort study,	
Aly-Khan A. Lalani (14)	2019	146	NK	PD-1, PD-L1	(−56,28)	Retrospective cohort study	
Amit A Kulkarni (15)	2020	195	Caucasian, African, American, Others	Nivolumab Pembrolizumab Others	(−28,42)	Retrospective cohort study	
Andrew F. Nyein (16)	2022	256	American	PD-1, PD-L1, CTLA-4	(−60,30)	Retrospective cohort study	
Angelo Castello (17)	2021	50	Italy	PD-1,PL-L1	(−30,30)	RCT	
Anne Schett (18)	2020	218	Switzerland	PD-1,PD-L1	(−60,30)	Retrospective cohort study	
Arielle Elkrief (19)	2019	74	NK	PD-1, CTLA-4	(0,30)	Retrospective cohort study	
Bertrand Routy (20)	2022	100	NK	PD-1,PL-L1	NK	RCT	
C hogue (21)	2019	161	American	PD-1	(−90,0)	Retrospective cohort study	
Coureche Kaderbhai (22)	2017	74	France	PD-1	(−90,0)	Retrospective cohort study	
David J. Pinato (23)	2019	196	London	PD-L1	(−30,0)	RCT	
Deniz Can Guve (24)	2021	93	Turkey	PD-1	(−90,90)	Retrospective cohort study	
F. Barroín (25)	2019	140	Mexico	PD-L1	(0,30)	Retrospective cohort study	
Florian Huemer (26)	2019	142	Austria	PD-1, PD-L1	(−30,30)	Retrospective cohort study	
Florian Huemer (27)	2018	30	Austria	PD-1	(−30,30)	Retrospective cohort study	
Hyunho Kim (28)	2019	234	Korea	CTLA-4, PD-1,PD-L1	(−60,0)	Retrospective cohort study	
Jahan J. Mohiuddin (29)	2020	568	American	PD-1, CTLA-4	(−90,90)	Retrospective cohort study	
Jhe-cyuan Guo (30)	2019	49	Taiwan	PD-1, PD-L1	(−60,30)	Retrospective cohort study	
Jibran Ahmed (31)	2018	60	USA	PD-1,PD-L1	(−14,14)	Retrospective cohort study	
Julia Ouaknine Krief (32)	2019	72	France	PD-1	(−60,30)	Retrospective cohort study	
Jwa Hoon Kim (33)	2021	53	Korea	PD-1	(−30,0)	Retrospective cohort study	
Ka Shing Cheung (34)	2021	412	China	PD-1, CTLA-4	(−30,30)	Retrospective cohort study	
Katharina Pomej (35)	2021	206	Vienna	NK	(−30,0)	Retrospective cohort study	
Kazuyuki Hamada (36)	2021	69	Japan	PD-1	(−21,21)	Retrospective cohort study	
Kosuke Ueda (37)	2019	31	Japan	PD-1, CTLA-4	(−30,0)	Retrospective cohort study	
L. Derosa (38)	2018	121	America	PD-L1	(−60,0)	Retrospective cohort study	
Laura M. Chambers (39)	2021	101	USA	PD-L1	(−30,0)	Retrospective cohort study, RCT	
Louis Gaucher (40)	2021	372	France	PD-1, CTLA-4	(0,60)	Retrospective cohort study	
M. Chalabi (41)	2020	1,512	Netherlands	PD-L1	(−30,30)	Retrospective cohort study,	
Megan Greally (42)	2019	161	American	PD-1,PD-L1, CTLA-4	(−60,0)	NK	
Metges (43)	2018	798	NK	PD-1	(−60,30),(−60,150)	Retrospective cohort study	
Min Jung Geum (44)	2021	140	NK	PD-1	NK	Retrospective cohort study	
Nadina Tinsley (45)	2020	347	England	NK	(−14,42)	Retrospective cohort study	
Nobuaki Ochi (46)	2021	531	Japan	PD-L1	(−60,60)	Retrospective cohort study	
Petros Fessas (47)	2021	450	Europe, North America, Asia	PD-1,PD-L1	(−30,0)(0,30)(−30,30)	Retrospective cohort study	
Pierre-Yves Cren (48)	2020	1,585	France	CTLA-4	(−60,60)	Retrospective cohort study	
Po-Hsien Lu MS (49)	2020	340	Taiwan	PD-1, PD-L1,CTLA-4	(−30,0)	Retrospective cohort study	
Quentin (50)	2021	212	France	PD-1	(−60,0)	Retrospective cohort study	
Sha Zhao (51)	2019	109	China	PD-1/PD-L1	(−30,30)	Retrospective cohort study	
Steven R. Hwang (52)	2020	62	USA	PD-1, CTLA-4	(−90,0)(0,90)	Retrospective cohort study	
Taiki Hakozaki (53)	2020	70	Japan	PD-1/PD-L1	(−30,0)	RCT	
Uqba Khan (9)	2021	414	American	PD-1,PD-L1, CTLA-4	(−84,84)	Retrospective cohort study	
X. Mielgo Rubio (54)	NK	121	Spanish	PD-1	(−60,60)	Retrospective cohort study	
Ying Jing (55)	2022	767	china	PD-1, PD-L1	(−90,90)	Retrospective cohort study	

NK, not known.

### Analysis of ATB use and clinic features

In this study, we performed a meta-analysis between ATB use and clinic features, including ECOG score (≤), PD-L1 expression (<1%), non-small-cell lung cancer (NSCLC) patients, gender (male) and ICI therapy line (0–1 prior), which were shown in **Supplementary Material**. However, we did not observe a clear significant association of these factors (p >0.05), except for the ECOG score (≤1). Among all the eligible studies, 20 studies were chosen to analyze the relationship between ATB use in ICI treatment and ECOG scores. The results showed that the cancer patients with ATB use in the clinic were associated with a lower ECOG score (≤1) in importance significance (HR: 0.69, 95% CI: 0.49–0.98, p = 0.04).

### The association between ATB use and survival outcomes (OS + PFS)

**Figure 2:**
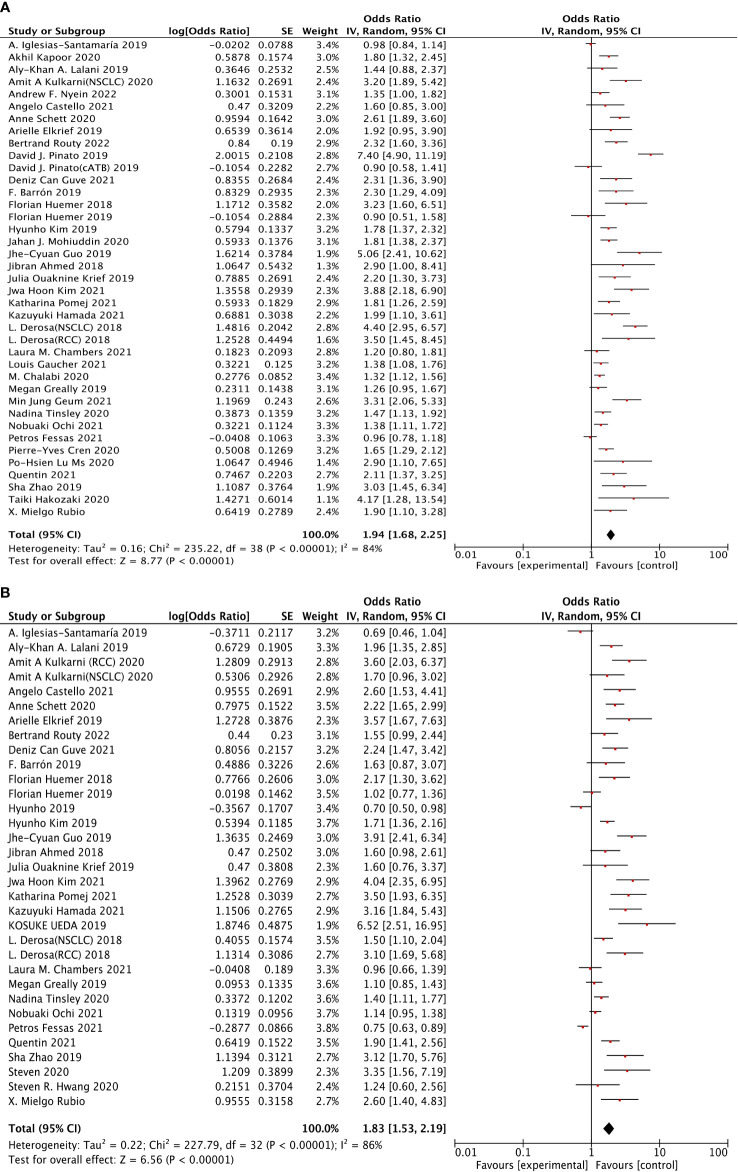
The forest plot showing the relationship between ATB use and OS, PFS in cancer patients treated with ICIs. Overall survival (OS), progress-free survival (PFS); CI, confidential interval; Random, random-effects model; The random-effects model was adopted. **(A)** Overall survival (OS). **(B)** Progress-free survival (PFS). **(A)** Relationship between ATB use and OS in cancer patients treated with ICIs. **(B)** Relationship between ATB use and PFS in cancer patients treated with ICIs.

Thirty-seven studies were selected to analyze overall survival (OS). The results revealed that ATB use was strongly associated with the increased risk of poor OS (HR: 1.94, 95% CI: 1.68–2.25, p <0.00001), shown in **Figure 2A**. However, a clear heterogeneity was observed in this analysis (I^2^ = 84%). Moreover, 31 studies were selected to perform progression-free survival (PFS). The results shown in **Figure 2B** indicated that ATB use was significantly associated with worse PFS (HR: 1.83, 95% CI: 1.53–2.19, p <0.00001), but also with an obvious heterogeneity (I^2^ = 86%).

### Sensitivity analysis

**Figure 3:**
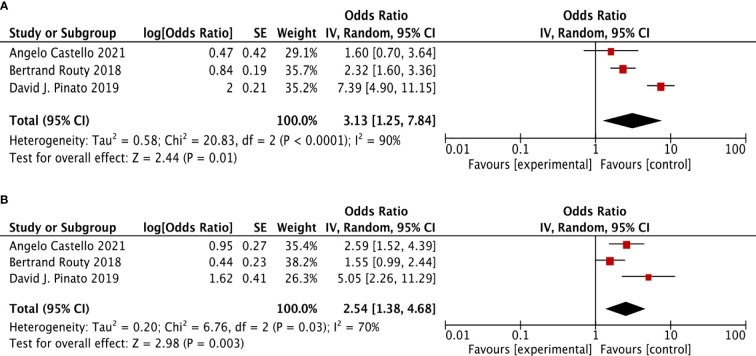
The forest plot showing the relationship between ATB use and OS, PFS in cancer patients treated with ICIs, based on randomized controlled trial (RCT). Overall survival (OS), progress-free survival (PFS); CI, confidential interval; Random, random-effects model. The random-effects model was adopted. **(A)** Overall survival (OS). **(B)** progress-free survival (PFS).

For further verification to identify the association between ATB use and the survival outcomes (OS + PFS) in ICI-treated cancer patients, we performed the same analysis in randomized controlled trial studies as the sensitivity analysis. Three RCT studies were selected. It revealed a similar result as above, that in cancer patients treated with ICIs, ATB use was significantly related to poor OS (HR: 3.13, 95% CI: 1.25–7.84, p <0.001, I^2^ = 90%) and poor PFS (HR: 2.54, 95% CI: 1.38–4.68, p <0.001, I^2^ = 70%) (**Figure 3**).

### In NSCLC, RCC, HCC, EC, and MEL, how does the ATB use impact the prognosis (OS + PFS) in patients treated in ICIs?

**Figure 4:**
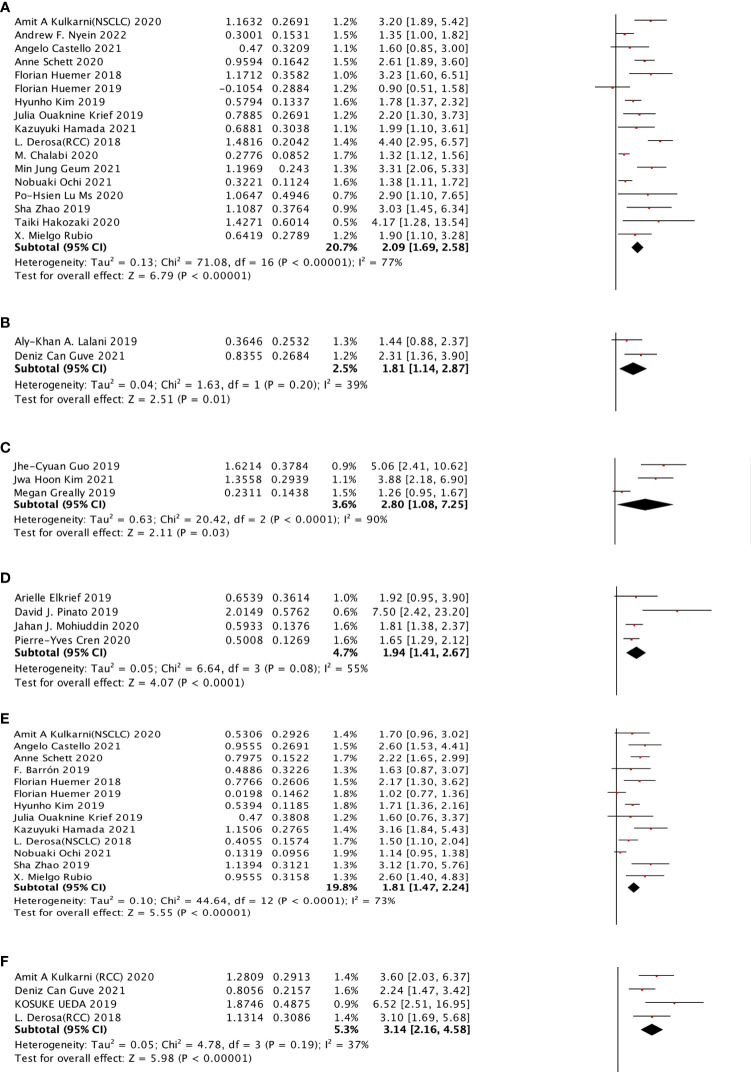
The subgroup analysis between ATB use and cancer prognosis (OS + PFS) of RCC and NSCLC cancer patients treated with ICIs. **(A)** The relationship between ATB use and OS of NSCLC patients treated with ICIs. **(B)** The relationship between ATB use and PFS of RCC patients treated with ICIs. **(C)** The relationship between ATB use and OS of esophagus cancer patients treated with ICIs. **(D)** The relationship between ATB use and OS of melanoma patients treated with ICIs. **(E)** The relationship between ATB use and PFS of NSCLC patients treated with ICIs. **(F)** The relationship between ATB use and PFS of RCC patients treated with ICIs.

For cancer types, we chose NSCLC, RCC, HCC, EC, and MEL to observe. In the OS sub-group analysis, seventeen studies were selected for NSCLC, two studies were selected for RCC, two studies were selected for HCC, three studies were selected for EC, and four studies were selected for MEL. NSCLC (HR: 2.09, 95% CI: 1.69–2.58), RCC (HR: 1.81, 95% CI: 1.14–2.87), EC (HR: 2.80, 95% CI: 1.08–7.25), and MEL (HR: 1.94, 95% CI: 1.41–2.67) were shown to be strongly associated with poor OS. However, no significant relationship was observed for HCC. Moreover, four different cancer types were included in the PFS subgroup analysis, including NSCLC, RCC, HCC, and EC, which indicated that NSCLC (HR: 1.81, 95% CI: 1.47–2.24) and RCC (HR: 3.14, 95% CI: 2.16–4.58) cancer types were associated with poor PFS with a strong effect and HCC, whereas EC was not significantly related (**Figure 4**).

### In cancer patients treated in PD-1 or PD-L1 ICI type, how does the ATB use impact the prognosis (OS + PFS)?

**Figure 5:**
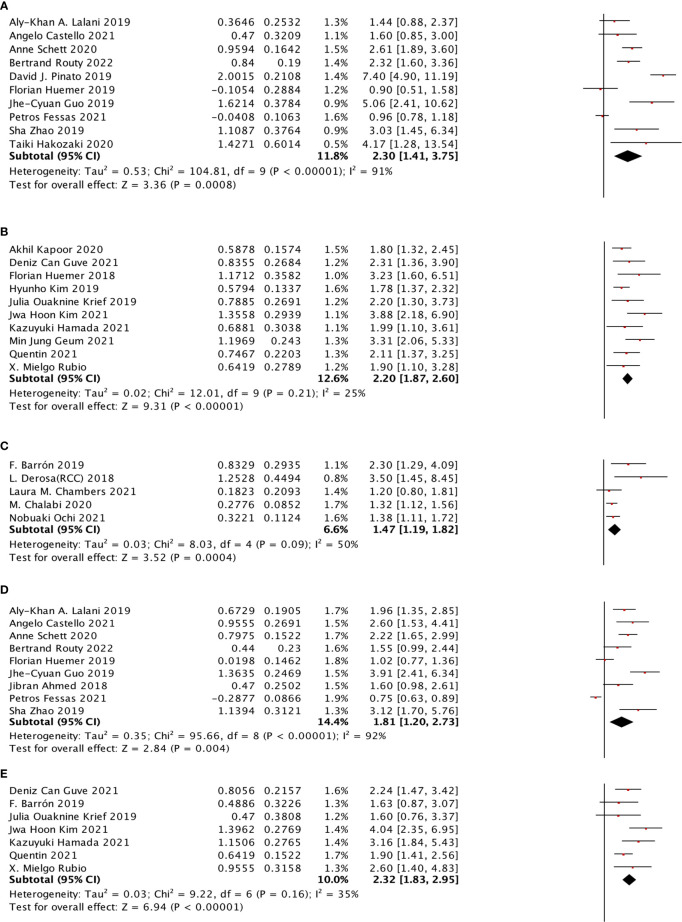
The subgroup analysis between ATB use and different immune checkpoint inhibitors of cancer patients treated with ICIs. **(A)** The association between ATB use and OS in cancer patients treated with the combination of PD-1 inhibitor and PD-L1 inhibitor. **(B)** The association between ATB use and OS in cancer patients treated with PD-1 inhibitor. **(C)** The association between ATB use and OS in cancer patients treated with PD-L1 inhibitor. **(D)** The association between ATB use and PFS in cancer patients treated with the combination of PD-1 inhibitor and PD-L1 inhibitor. **(E)** The association between ATB use and PFS in cancer patients treated with PD-1 inhibitor.

PD-1 inhibitor, PD-L1 inhibitor, and the combination of PD-1 inhibitor and PD-L1 inhibitor were selected to do the sub-analysis for ICI type. The results showed that all the three types showed a stronger effect on OS (PD-1 inhibitor: HR: 2.20, 95% CI: 1.87–2.60, p <0.00001, I^2^ = 25%; PD-L1 inhibitor: HR: 1.47, 95% CI: 1.19–1.82; combination of PD-1 inhibitor and PD-L1 inhibitor: HR: 2.30, 95% CI: 1.41–3.75). Meanwhile, the same inhibitor types were observed in the PFS sub-analysis, and only the PD-1 inhibitor (HR: 2.32, 95% CI: 1.83–2.95) and the combination of PD-1 inhibitor and PD-L1 inhibitor (HR: 1.81, 95% CI: 1.20–2.73) showed a significant relationship with PFS (**Figure 5**).

### What is the relationship between ATB use and survival outcome (OS + PFS) of patients according to different ATB windows?

**Figure 6:**
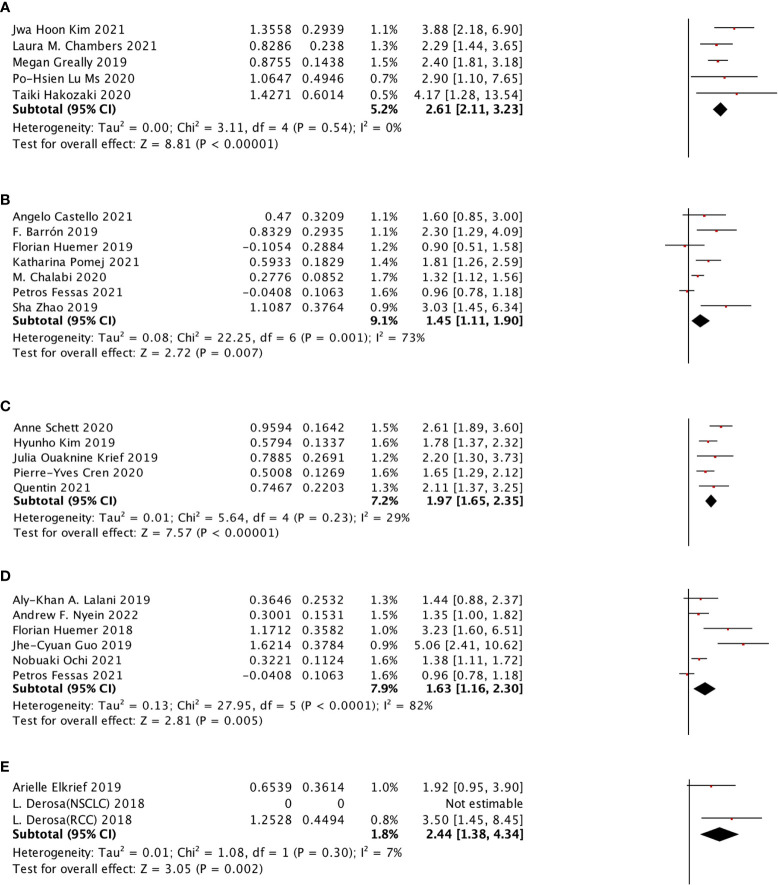
In different ATB windows, the subgroup analysis between ATB use and OS of cancer patients treated with ICIs. **(A)** ATB window (−30 days, 0 day); **(B)** ATB window (−30 days, 30 days); **(C)** ATB window (−60 days, 0 days); **(D)** ATB window (−60 days, 30 days); and **(E)** ATB window (0 days, 30 day).

The selected ATB window included (−60 days, +30 days), (−60 days, 0 day), (−30 days, 30 days), (−30 days, 0 day), (0 day, +30 days) for OS subgroup analysis and (−60 days, +30 days), (−60 days, 0 day), (−30 days, 30 days), (0 day, +30 days) were selected for PFS subgroup analysis. All of these groups were shown to be significantly associated with poor survival outcomes. We also performed the PFS subgroup analysis for using ATB treatment during ICI treatment, and no significant relation was observed (**Figure 6**).

### In broad-spectrum ATB class, the relationship of ATB use and PFS

**Figure 7:**
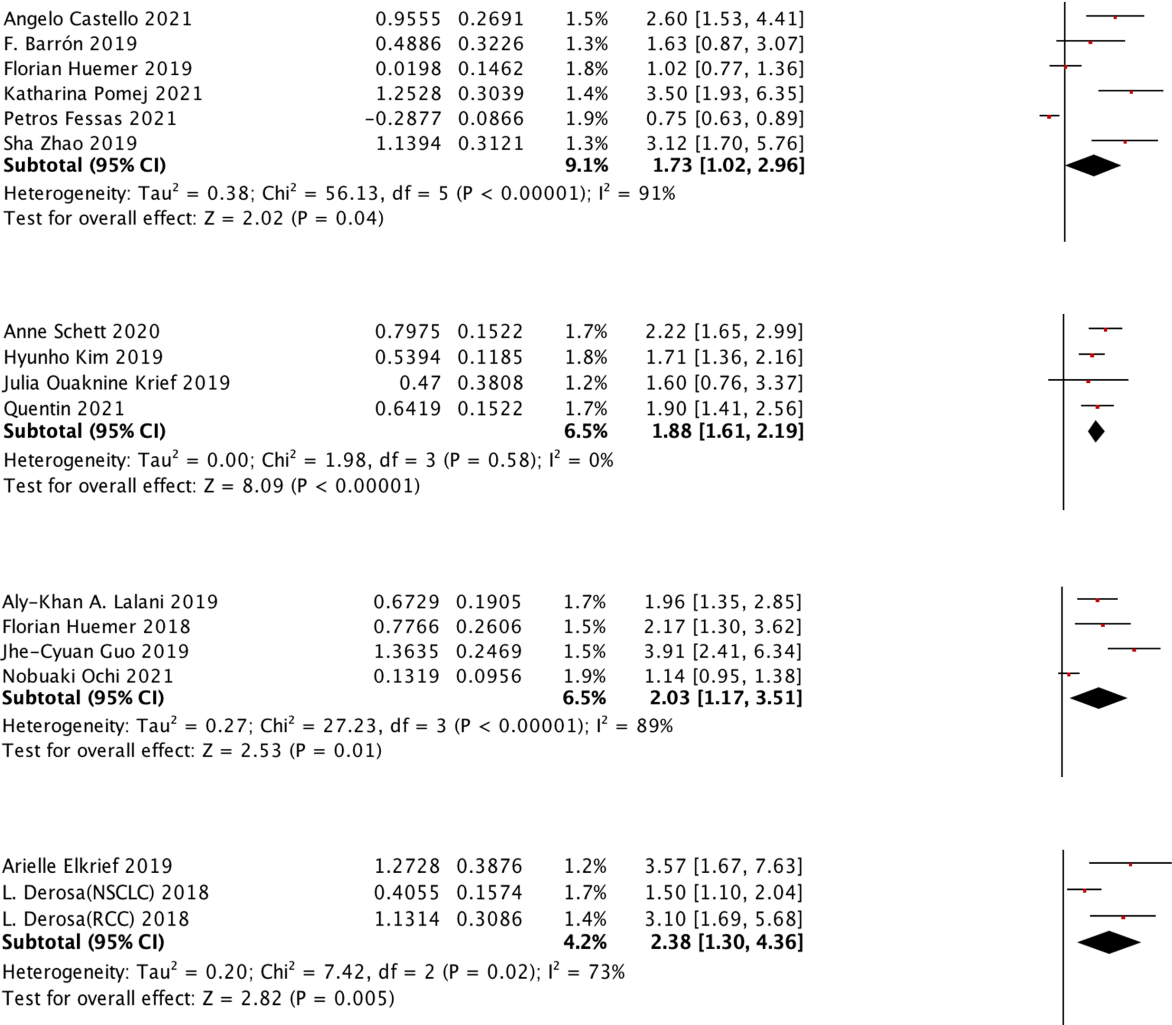
In different ATB window, the subgroup analysis between ATB use and PFS of cancer patients treated with ICIs. **(A)** ATB window (-30 days, 30 day); **(B)** ATB window (-60 days, 0 days); **(C)** ATB window (-60 days, 30 days); **(D)** ATB window (0 days, 30 days).

**Figure 8:**
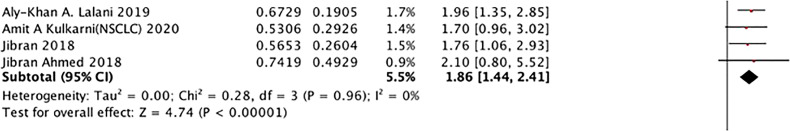
In broad- spectrum ATB class, the subgroup analysis between ATB use and PFS of cancer patients treated with ICIs.

The analysis between the use of broad-spectrum ATB class and PFS of ICI treated cancer patients was also performed, as shown in **Figure 7**, which was the first mentioned in this research. The result, with no heterogeneity (I^2^ = 0), revealed that this class was strongly related to poor PFS (HR: 1.86, 95% CI: 1.44–2.41) **Figure 8**.

### ECOG score and OS

No significant difference was observed between higher ECOG score and OS, compared with lower ECOG score (≤1) (HR: 0.49, 95% CI: 0.09–2.76, p = 0.42).

### Assessment of publication bias

The publication bias for this research was evaluated by funnel plots, which were collected and shown in the **Supplementary Material**. There was no obvious publication bias in this research. Newcastle-Ottawa scale scores from 6 to 9 (**Table 2**). The heterogeneity value also indicated a low publication bias (**Table 3**).

**Table 2:** Basic characteristics of the studies included in the meta-analysis (n = 51).

Study	Cancer type	Median PFS (ATB vs non-ATB)	Median OS (ATB vs non-ATB)	NOS score
A. Iglesias‐Santamariía (12)	locally advanced/metastatic cancer	4.3 months vs. 5.8 months	11.7 months vs. 14.5 months,	7
Akhil Kapoor (13)	Lung cancer, head and neck cancer, others	3.6 months vs 1.7 months	3.9 months vs 9.2 months	6
Aly-Khan A. Lalani (14)	mRCC	7.2 months vs NK	12.0 months vs NK	7
Amit A Kulkarni (15)	NSCLC, RCC, AML	1.5 months vs 4.0 months	3.0 months vs 12.0 months	7
Andrew F. Nyein (16)	NSCLC	NK	NK	6
Angelo Castello (17)	NSCLC	4.1 months vs 12.4 months	11.3 months vs 15.3 months	8
Anne Schett (18)	NSCLC	1.9 months vs 3.8 months	7.9 months vs 23.6 months	8
Arielle Elkrief (19)	melanoma	2.4 months vs 7.3 months	7.5 months vs 18.3 months	8
Bertrand Routy (20)	NSCLC, RCC, urothelial carcinoma	3.5 months vs 4.1 months	11.5 months vs 20.6 months	8
C Hogue (21)	NSCLC	NK	NK	6
Coureche Kaderbhai (22)	NSCLC	NK	NK	7
David J. Pinato (23)	Primary lung, Clear cell renal cell carcinoma, Primary head and neck squamous cell carcinoma Malignant melanoma, Transitional cell carcinoma	NK	14.6 months vs NK	7
Deniz Can Guve (24)	RCC	23.75 ± 4.41 months	8.44 ± 1.61 months	8
F. Barroín (25)	NSCLC	1.9 months vs 2.7 months	2.04 months vs 12.42 months	9
Florian Huemer (26)	NSCLC	3.8 months vs 4.0 months	14.6 months vs 11.2 months	8
Florian Huemer (27)	NSCLC	2.9 months vs 3.1 months	7.5 months vs 15.1 months	9
Hyunho Kim (28)	Non-small-cell lung carcinoma	2 months vs 4 months	5 months vs 17 months	8
Jahan J. Mohiuddin (29)	melanoma	NK	27.4 months vs 43.7 months	7
Jhe-cyuan Guo (30)	ESCC	1.3 months vs 2.8 months	3.0 months vs 10.4 months	8
Jibran Ahmed (31)	Lung cancer, Renal cancer Hepatocellular cancer Head and neck cancer Urothelial cancer Malignant melanoma	NK	24 weeks vs 89 weels	7
Julia Ouaknine Krief (32)	non-small cell lung cancer	1.8 months vs 3 months	5.1 months vs 13.3 months	9
Jwa Hoon Kim (33)	Esophageal squamous cell carcinoma	1.9 months vs NK	6.4 months vs NK	8
Ka Shing Cheung (34)	hepatocellular carcinoma	NK	NK	7
Katharina Pomej (35)	HCC	3.5 months vs 4.8 months	4.7 months 11.4 months	8
Kazuyuki Hamada (36)	NSCLC	NK	8.12 months vs 28.7 months	8
Kosuke Ueda (37)	RCC	2.8 months vs 18.4 months	NK	8
L. Derosa (38)	NSCLC,RCC	1.9 months vs 7.4 months	17.3 months vs 30.6 months	9
Laura M. Chambers (39)	Endometrial carcinoma Cervical carcinoma; Cvarian carcinoma	7.3 months vs NK	11.6 months vs NK	7
Louis Gaucher (40)	Lung, Melanoma, Renal and urothelial, Head and neck, Hodgkin’s lymphoma, Digestive, Cutaneous squamous cell carcinoma, Adenocarcinoma of unknown primary, Squamous cell carcinoma of unknown, Porocarcinoma	43.0 months vs 96.9 months	36.1 months vs 86.3 months	9
M. Chalabi (41)	NSCLC	NK	8.5 months vs 11.0 months	7
Megan Greally (42)	Advanced Esophagogastric Cancer	1.2 months vs 1.8 months	2.0 months vs 6.4 months	8
Metges J (43)	malignant melanoma and lung cancer	NK	NK	6
Min Jung Geum (44)	NSCLC	NK	NK	7
Nadina Tinsley (45)	melanoma, non-small cell lung cancer, renal cell carcinoma	3.1 months vs 6.3 months	10.4 months vs 21.7 months	8
Nobuaki Ochi (46)	nonesmall-cell lung cancer	3.5 months vs 3.5 months	11.7 months vs 16.1 months	8
Petros Fessas (47)	HCC	4.4 months vs 7.2 months	15.4 months vs 16.4 months	7
Pierre-Yves Cren (48)	advanced melanoma	7.3 months vs 2.4 months	15.4 months vs 14.5 months	8
Po-Hsien Lu MS (49)	NSCLC	8.87 months vs 15.17 months	4.03 days vs 12.3 months	7
Quentin (50)	non-small cell lung carcinoma, melanoma, upper airway carcinoma, digestive tract carcinoma renal cell carcinoma	NK	NK	6
Sha Zhao (51)	NSCLC	3.7 months vs 9.6 months	6 months vs 21.9 months	8
Steven R. Hwang (52)	Hodgkin lymphoma	NK	NK	6
Taiki Hakozaki (53)	NSCLC	5.2 months vs NK	16.2 months vs NK	7
Uqba Khan (9)	NK	NK	NK	6
X. Mielgo Rubio (54)	NSCLC	NK	NK	6
Ying Jing (55)	Lung cancer, Liver cancer, Esophageal cancer, Head and neck cancer, Cholangiocarcinoma, Cervical cancer, Lymphoma, Sarcoma, Other	NK	NK	6

NK, not known.

**Table 3:** Subgroup analysis of ECOG, cancer type, ICI type, and ATB window based on OS (overall survival) and PFS (progress-free survival).

Subgroup		OS											PFS						
		(OR, 95%CI)	No. of studies	No of patients	*p*	Heterogeneity					(OR, 95%CI)	No. of studies	No. of patients	*p*	Heterogeneity				
			Tau^2^			Chi^2^	df	*I2*(%)	p					Tau^2^	Chi^2^	df	*I2*(%)	p
ECOG		0.94 (0.33, 2.66)	3	654	0.91	1.12	24.19	2	92	<0.001									
Cancer type	NSCLC RCC	2.09 (1.69, 2.58) 1.81 (1.14, 2.87)	17 2	4,155 239	<0.001 0.01	0.13 0.04	71.08 1.63	16 1	77 39	<0.001 0.2	1.81 (1.47, 2.24) 3.14 (2.16, 4.58)	13 4	2,032 440	<0.001 <0.001	0.1 0.05	44.64 4.78	12 3	73 37	<0.001 0.19
HCC	1.30 (0.70, 2.41)	2	655	0.41	0.18	8.98	1	89	0.003	1.58 (0.35, 7.13)	2	655	0.55	1.14	23.77	1	96	<0.001
Esophageal cancer	2.80 (1.08, 7.25)	3	270	0.03	0.63	20.42	2	90	<0.001	2.54 (0.96, 6.69)	3	270	0.06	0.18	31.78	2	94	<0.001
	Melanoma	1.94 (1.41, 2.67)	4	2,441	<0.001	0.05	6.64	3	55	0.08									
ICIs type	PD-1 inhibitor PD-L1 inhibitor	2.20 (1.87, 2.60) 1.47 (1.19, 1.82)	10 5	1,312 1,062	<0.001 <0.001	0.02 0.03	12.01 8.03	9 4	25 50	0.21 0.09	2.32 (1.83, 2.95) 1.42 (0.95, 2.13)	7 4	767 893	<0.001 0.09	0.03 0.12	9.22 12.02	6 3	35 75	0.16 0.007
	PD-(L)1 inhibitor	2.30 (1.41, 3.75)	10	1,678	<0.001	0.53	104.81	9	91	<0.001	1.81 (1.20, 2.73)	9	1,332	0.004	0.35	95.66	8	92	<0.001
ATB window (−30,0) (−30,30) (−60,0)		2.61 (2.11, 3.23) 1.45 (1.11, 1.90) 1.97 (1.65, 2.35)	5 7 5	732 2,608 2,447	<0.001 0.007 <0.001	0 0.08 0.01	3.11 22.25 5.64	4 6 4	0 73 29	0.54 0.001 0.23	1.73 (1.02, 2.96) 1.88 (1.61, 2.19)	6 4	1,096 703	0.04 <0.001	0.38 0	56.13 1.98	5 3	91 0	<0.001 0.58
(−60,30) (0,30) during		1.63 (1.16, 2.30) 2.44 (1.38, 4.34)	6 3	1,461 269	0.005 0.002	0.13 0.01	27.95 1.08	5 1	82 7	<0.001 0.3	2.03 (1.17, 3.51) 2.38 (1.30, 4.36) 1.07 (0.53, 2.15)	4 3 3	756 195 397	0.01 0.005 0.85	0.27 0.20 0.32	27.23 7.42 14.58	3 2 2	89 73 86	<0.001 0.02 <0.001
Broad-spectrum ATB			3								1.86 (1.44, 2.41)	3	255	<0.001	0	0.28	3	0	0.96

## Discussion

This research is the most comprehensive study on the effect of antibiotic use on the clinical features and survival outcomes of cancer patients treated with ICIs, compared with previous meta-analysis until now. In this meta-analysis, from 45 studies with 12,493 patients, the effects of ATB use on OS, PFS, and clinical features were included to study the impacts of ATB use on cancer patients treated with ICI therapy, with RCT analysis as verification. Based on OS analysis and PFS analysis, we performed several subgroup analyses from 5 aspects, cancer type (NSCLC, RCC, HCC, EC, and MEL), ICI therapy type (PD-1, PD-L1), ATB window (−60 days, +30 days), ATB class (broad-spectrum ATB class) and ECOG score (2–5 vs 0–1).

Our findings revealed that the ATB use was related with worse OS and PFS, which was similar with previous study (6). ATB treatment is commonly performed in clinic for cancer patients, who are more susceptible to getting infected, but the ATBs can alter the composition and diversity of the gut microbiota. Therefore, ATB use can significantly impact the efficacy of ICIs. In subgroup analysis, for various cancer types, we analyzed non-small-cell lung cancer (NSCLC), renal cell carcinoma (RCC), hepatocellular carcinoma (HCC), esophageal cancer (EC), and melanoma (MEL). All of the five cancer types were shown to be at a higher risk of poor OS except HCC, while only NSCLC and RCC were shown to be at a higher risk of poor PFS. Among all the five types, EC was at the highest risk (HR = 2.8) in OS analysis, and RCC was at the highest risk in PFS analysis, even higher than 3 (HR = 3.14). Interestingly, in PFS analysis, no significant association was observed between EC and PFS of cancer patients treated in ICIs (p = 0.06), with only three eligible studies and high heterogeneity, which could be a focus of future research. HCC was shown not to be significantly associated with both OS and PFS, but with only two studies, which needs more studies for further verification. Various cancer types have different impacts on the human body. For the gut environment, a favorable gut microbiota can enhance antigen presentation and T-cell function related to the systemic and anti-tumor immune response, which was demonstrated in a mouse experiment (7). The diversity of gut microbiota increases from infancy to adulthood and decreases in the elderly, with metabolic, defensive, and trophic functions (56). Induction and regulation of the adaptive immune system is one of the essential aspects of the gut microbiota trophic function, and intestinal immunity is the largest and most complex part of the overall immune system of the human body, with at least 80% of all antibodies produced in the intestinal mucosa for adults (57). Thus, ATB use may reduce the efficacy of ICI immunotherapy through altering the diversity and composition of the gut microbiota, which still needs more evidence to prove.

For ICI therapy type, we selected PD-1 inhibitor type, PD-L1 inhibitor type, and the combination of both PD-1 inhibitor and PD-L1 inhibitor. The results revealed that the PD-1 inhibitor and the combination were strongly associated with a higher risk of poorer prognosis, while PD-L1 was shown to be out of meaningful relationship with PFS. Interestingly, we found that the HR value of the combination was quite lower than the HR value of the PD-1 inhibitor alone, which may indicate that the PD-L1 inhibitor matters a lot in this process. Rounis and his team analyzed 66 patients who received PD-1 inhibitors or PD-L1 inhibitors and found that ATB administration did not affect the survival outcome of ICI patients, but prolonged ATB use was related to poor survival (58). This contradiction may be attributed to different varieties, such as the amount of study population, cancer type, and ATB type. In our research, it was indicated that different ATB windows had effects on the survival outcome of ICI patients, when the ATB window was in the period between 60 days before ICI initiation and 30 days after ICI initiation. Especially when ATB window was (−30 days,0 day) of ICI initiation, the risk was the highest in OS analysis (HR = 2.61), with no heterogeneity (I2 = 0). When ATB window was (0 day, +30 days) of ICI initiation, the risk was the highest in the PFS analysis (HR = 2.38). Some studies have already revealed that the short-term decrease in bacterial richness after treatment in ATB (59). Meanwhile, the status of gut microbiota can recover to a baseline within 3 months after ATB discontinuation (60). So, using ATB treatment is essential, which can significantly influence the survival outcome of cancer patients on ICI therapy. Also, we also found the relationship of clinic feature, it was revealed that patients with a lower ECOG score (≤1) were more pretended to undergo ATB treatment. While the other aspects (PD-1 inhibitor type, NSCLC, gender type, cancer stage, and ICI line therapy) were observed to have no significant association.

Until now, the concrete mechanisms of how the use of antibiotics can impact the ICI therapy efficiency for cancer patients are still unknown, but some studies have shown that it may also be associated with the tumor microenvironment (61). An intact commensal microbiota is necessary for cancer therapy, which can mediate therapy effects through modulating the myeloid-derived cell functions in the tumor microenvironment. For example, in one experiment with ATB-treated mice, the tumor-infiltrating myeloid-derived cells responded poorly to the therapy, leading to lower cytokine production and tumor necrosis after CpG-oligonucleotide treatment, and it also showed deficient production of reactive oxygen species (ROS) and cytotoxicity after chemotherapy (62). Another research has indicated that ATB may change the equilibrium of commensal bacteria, conducive for ICB efficacy, which may result in possible resistance to ICIs (63). Meanwhile, the local microbiota was demonstrated to make up an important part of the tumor microenvironment in many types of cancer, which may be affected by ATB use (64). Many researchers have proved that local bacterial dysbiosis can cause a pro-inflammatory immune response and thereby promote cancer growth (65).

Compared with the previous meta-analysis, our research is the most comprehensive, which included the largest number of studies, the largest population, and studied the most comprehensive aspects of subgroup analysis. Yu et al. (66) and Jiang et al. (67) performed a similar meta-analysis, although their subgroup was not as comprehensive as ours. Lurienne et al. (68) and Chen et al. (69) only included NSCLC patients with great limitations. The research by Elkrief (70) missed relative statistical analysis. However, there are still several limitations to our current study. First, the heterogeneity of the included research cannot be ignored. Different responses to drugs, different intervals of administration, and different individual cancer status can result in high heterogeneity. Second, the included studies did not provide enough details. Although we recorded the baseline characteristics of the population and performed the subgroup analysis, some concrete aspects are still unclear, such as infection type and infection site. The subgroup analysis for the ICI type lacked CTLA-4 inhibitor, which was inadequate. Thirdly, most of the studies were retrospective, and only five of the studies contained randomized controlled trials.

## Conclusion

In this research, it was revealed that ATB use was strongly associated with worse OS and PFS in cancer patients treated with ICI immunotherapy, especially during the period between 60 days before ICI initiation and 30 days after ICI initiation, which indicated that ATBs should be used cautiously and strictly to avoid a worse survival outcome. The immunotherapy inhibitor type and ATB class can also impact the prognosis. Moreover, it was found that different cancer types are also essentially associated with a survival outcome, including NSCLC, RCC, EC, and MEL. Still, more studies are needed to find the concrete mechanism between ATB use and ICIs and further improve the clinical treatment.

## Data availability statement

The original contributions presented in the study are included in the article/**Supplementary Material**. Further inquiries can be directed to the corresponding authors.

## Author contributions

JZ conceived the project and wrote the manuscript. GH, W-CW, D-hH, J-wZ, and HZ participated in data analysis. HT participated in language editing. RL and HZ reviewed the manuscript. All authors contributed to the article and approved the submitted version.

## Funding

This work was partially supported by the Guangdong Province Medical Science and Technology Research Fund Project (A2021056), funded by the Special Fund for Scientific Research and Cultivation of Shunde Hospital Affiliated to Jinan University (202101011); and the Guangdong Innovation and Entrepreneurship Training Program for Undergraduate (S202110559091X, S202210559078).

## Acknowledgments

We would like to thank Taiwen Li and Shirley Liu from Harvard University for helpful suggestions.

## Conflict of interest

The authors declare that the research was conducted in the absence of any commercial or financial relationships that could be construed as a potential conflict of interest.

## Publisher’s Note

All claims expressed in this article are solely those of the authors and do not necessarily represent those of their affiliated organizations, or those of the publisher, the editors and the reviewers. Any product that may be evaluated in this article, or claim that may be made by its manufacturer, is not guaranteed or endorsed by the publisher.
