# Supplementary material for: The impact of antibiotic use on clinical features and survival outcomes of cancer patients treated with immune checkpoint inhibitors
Source: Front Immunol. 2022 Jul 28;13:968729. doi: 10.3389/fimmu.2022.968729 (PMC9367677; doi:10.3389/fimmu.2022.968729)
Supplement: Supplementary file 1 [file DataSheet_1.docx]

Supplementary figure 1: the relationship between ATB clinical use and different clinic features of cancer patients treated in ICIs. a. ECOG score (0-1), b. gender type (male), c. PD-L1 expression < 1%, d. cancer stage, e. NSCLC cancer type, f. line therapy (0-1 prior)

Supplementary figure 2: the funnel group of analysis. a. OS outcome analysis, b. PFS outcome analysis, c. OS outcome subgroup analysis, d. PFS outcome subgroup analysis

Supplementary figure 3: a. the relationship between OS and ECOG score (2-5 vs 0-1), b. the relationship between OS and NSCLC cancer type, c. the relationship between PFS and ATB window of (-30,30), d. the relationship between PFS and PD-1 ICIs type.
